# Adaptive Elements in Internet-Delivered Psychological Treatment Systems: Systematic Review

**DOI:** 10.2196/21066

**Published:** 2020-11-27

**Authors:** Suresh Kumar Mukhiya, Jo Dugstad Wake, Yavuz Inal, Ka I Pun, Yngve Lamo

**Affiliations:** 1 Western Norway University of Applied Sciences Bergen Norway; 2 NORCE Norwegian Research Centre Bergen Norway; 3 University of Bergen Bergen Norway

**Keywords:** cognitive behavioural therapy, internet-delivered psychological treatment, adaptive treatment, internet-based treatment, adaptive system, mental health, literature review, architecture centric development, tailored internet interventions, flexible mHealth internet interventions

## Abstract

**Background:**

Internet-delivered psychological treatments (IDPTs) are built on evidence-based psychological treatment models, such as cognitive behavioral therapy, and are adjusted for internet use. The use of internet technologies has the potential to increase access to evidence-based mental health services for a larger proportion of the population with the use of fewer resources. However, despite extensive evidence that internet interventions can be effective in the treatment of mental health disorders, user adherence to such internet intervention is suboptimal.

**Objective:**

This review aimed to (1) inspect and identify the adaptive elements of IDPT for mental health disorders, (2) examine how system adaptation influences the efficacy of IDPT on mental health treatments, (3) identify the information architecture, adaptive dimensions, and strategies for implementing these interventions for mental illness, and (4) use the findings to create a conceptual framework that provides better user adherence and adaptiveness in IDPT for mental health issues.

**Methods:**

The review followed the guidelines from Preferred Reporting Items for Systematic Reviews and Meta-Analyses (PRISMA). The research databases Medline (PubMed), ACM Digital Library, PsycINFO, CINAHL, and Cochrane were searched for studies dating from January 2000 to January 2020. Based on predetermined selection criteria, data from eligible studies were analyzed.

**Results:**

A total of 3341 studies were initially identified based on the inclusion criteria. Following a review of the title, abstract, and full text, 31 studies that fulfilled the inclusion criteria were selected, most of which described attempts to tailor interventions for mental health disorders. The most common adaptive elements were feedback messages to patients from therapists and intervention content. However, how these elements contribute to the efficacy of IDPT in mental health were not reported. The most common information architecture used by studies was tunnel-based, although a number of studies did not report the choice of information architecture used. Rule-based strategies were the most common adaptive strategies used by these studies. All of the studies were broadly grouped into two adaptive dimensions based on user preferences or using performance measures, such as psychometric tests.

**Conclusions:**

Several studies suggest that adaptive IDPT has the potential to enhance intervention outcomes and increase user adherence. There is a lack of studies reporting design elements, adaptive elements, and adaptive strategies in IDPT systems. Hence, focused research on adaptive IDPT systems and clinical trials to assess their effectiveness are needed.

## Introduction

Research accounts for internet-delivered psychological treatment (IDPT) as a useful therapeutic tool [1] with increased potential to provide evidence-based mental health interventions for the far-reaching population at a lower cost [2,3]. However, actual user adherence to such interventions is low [4-8]. These results raise a critical question in IDPT: how can the clinical effect of IDPT be improved? Therefore, it is relevant to focus on the factors associated with enhancing user adaption toward such interventions.

Some studies have found that providing therapist contact for online guidance and support during interventions increases adherence and effect sizes [7,9-14]. Clarke et al [15] added telephone calls and postcard reminders from therapists to increase user adherence. The study concluded by discovering no significant difference between intervention groups with or without reminders. However, a similar study done by Farrer et al [16] to evaluate the effectiveness of a 6-week IDPT for depression with and without telephone interaction concluded that IDPT is effective both with and without tracking for reducing depression. The results indicated that the success of an intervention depends on the environmental settings in which they are performed [16]. Similarly, some studies increased the frequency of emails from therapists to increase user adherence. Klein et al [17] conducted a study to examine if the frequency of therapists’ contact (from 1 email per week to 3 emails per week) made a difference in user adherence. The study concluded that the effectiveness of IDPT might be independent of the frequency of therapist support. Hilvert-Bruce et al [18] conducted a study to investigate if the drop out of users of IDPT was caused by lack of treatment efficacy, if changes in the choice of treatments, reminders, and financial cost improves adherence, and finally, if the addition of clinical contact improves user adherence. The results of the study showed that adding reminders, and increasing the choice of treatments, cost, timing, and contact with clinicians improved user adherence [18]. These findings illustrate that the baseline predictors of adherence vary across studies.

A systematic review by Christensen et al [7] discovered that disease diversity, treatment length, and predicted chronicity are essential factors contributing to user adherence in IDPT. Similarly, clinical severity has also been indicated as one of the crucial factors contributing to user adherence in web-based interventions targeting problematic drinking [19]. Similar factors have been identified as the most prominent factors in user adherence toward IDPT. However, only a few studies have discussed why the target group chose not to adhere to any specific IDPT system. The most common reasons cited for nonadherence in state-of-art studies were that (1) people believe they have made sufficient progress, (2) users reported there was too much content without much flexibility, (3) users reported that treatments were too complicated, (4) treatments did not match users’ expectations, (5) there was a lack of therapist contact, and (6) there was a lack of personalization. A meta-analysis by Vandereycken and Devidt [20] concluded that the target groups chose not to adhere to the eating disorder treatment because they believed they had achieved sufficient progress. However, a lack of progress is not related to nonadherence, according to several other studies [7,17]. According to a survey done by Johansson et al [21], participants chose not to adhere to the treatment when they were unable to perceive a compatible correlation between the length of weekly text modules and the conditions in their personal life. Moreover, the participants found the content to be a tiresome burden because of the length of the text modules and time consumed to go through them. Furthermore, the fixed format of the content sent to the participants each week was perceived as inflexible for some participants. Content complexity was perceived as challenging to comprehend and to process by individuals participating in interventions [8,21], especially when these individuals considered themselves to have attention problems or limited reading and writing skills. Participants‘ knowledge and expectations about the treatment process have shown to influence user trust and hence adherence [22]. Johansson et al [21] outlined in their study that participants mentioned they were grateful for being offered the treatment. However, not all of them appeared to be fully aware of the treatment and its significance. A similar conclusion was drawn by Alaoui et al [23], who identified higher treatment credibility to be the most influential prognostic factor for user adherence. Feedback has been thought to increase user adherence for 65% of intervention participants [19]. Similarly, a study by Johansson et al [21] revealed that the lack of therapist support during interventions was perceived by patients as a sign that therapists did not care about their health care issues. Furthermore, some participants reported that they never prioritized their personal development because they were aware that face-to-face meeting was not required. A recent study on mental health indicated that compliance failure can result from a lack of personalization [24]. A study by Doherty [25] claimed to have improved user adherence with the IDPT system by focusing on user personalization.

Most of the research examining the causes of low user adherence to IDPT has discovered that the reasons associated with patients were about personal and interpersonal competencies, and lack of resources rather than the diagnosis or health problem severity [7]. Moreover, it was about the patient’s intrinsic motivation to change, their self-relatedness, and their receptivity to change. Levey and Clarkin [26] characterized this reason as the patient variable. Considering this as the reason for premature termination of interventions indicates a need to investigate the reasons and circumstances for nonadherence further. Specifically, this indicates a gap in the literature concerning the in-depth exploration of the subjective reasons for nonadherence in online psychological interventions. In general, the factors affecting premature termination of participants from IDPT, as outlined by Johansson et al [21], can be characterized by the interaction between the participant’s perception of the treatment (content complexity, therapist feedback, and information about significance) and the participant’s situation (awareness about the treatment, availability, daily routines, treatment expectations, and perceived language skills). Analogously, a report by the World Health Organization [27] distinguished five interacting dimensions affecting adherence to medication, therapy, and health care in general: socioeconomic factors, therapy-related factors, patient-related factors, condition-related factors, and health system/health care team–related factors. The same report claimed that relatively limited research has been done on the effects of health system/health care team–related factors on adherence.

In this paper, we propose that in addition to these two factors (perception of treatment and personal situations), a third factor is contributing to user adherence: the adaptiveness of the IDPT system. There are two perspectives here: adaptiveness and information architecture (IA) [28]. First, IA is associated with how people cognitively process information and enhances the ability of the participants to find information. Second, adaptiveness refers to an ability in the system to change in response to environmental changes. The former perspective makes the information presented in IDPT comprehensible and discoverable, while the latter makes the IDPT more personalized. In this paper, we argue that both adaptiveness and IA are essential elements that contribute to user adherence in IDPT. Hence, we aim to investigate the following research questions in this literature review: (1) what are the most prevalent choices of IA in existing IDPT systems, and what is the primary rationale behind choosing an IA?, (2) what are the primary adaptive elements in IDPT systems, and how do these elements contribute to enhancing user adherence and intervention outcomes?, (3) what are the primary adaptive strategies used in IDPT systems, and how do these adaptive strategies consume adaptive elements to generate personalized experience for mental health patients?, and (4) how can we generalize the results to create a conceptual framework that can be used in the creation of an adaptive IDPT system for mental health interventions?

To the best of our knowledge, limited research has examined the experience of nonadherence in the IDPT system based on IA and adaptiveness as affecting factors. In this study, we focus on reviewing the adaptive elements and IA in the current IDPT systems used for the treatment of mental illness. Our review shows that several different terms are being used to describe similar IDPT systems. Interventions involving the internet as the communication mechanism are referred to as web-based treatments, web-based interventions, online treatment, computerized psychotherapy, e-therapy, eHealth, internet-based cognitive behavioral therapy, digital interventions, web app–based psychotherapy treatments, therapeutic web-based interventions, eHealth interventions [29], and others. Analogously, other variations include creation of technical platforms such as Interapy [30], Deprexis [31], ULTEMAT [32], digital behavior change interventions [33], and smartphone-based apps with specific brand names [34]. The absence of any taxonomic preferences and professional ontology makes the field of IDPT inconsistent and ambiguous. The use of a multitude of terms and labels to describe similar health interventions makes it difficult to search the results of studies. To be consistent, we chose to use the term IDPT, as suggested by Andersson et al [35].

## Methods

We conducted the review according to the PRISMA (Preferred Reporting Items for Systematic Reviews and Meta-Analyses) guidelines [36]. Here, we present the methodology we used to search, analyze, and extract pertinent information from relevant studies.

### Search Strategy

We searched the databases recommended by Cochrane [37], including Medline (PubMed), ACM Digital library, PsycINFO, EMBASE, CINAHL, and Cochrane, to identify studies. In addition, we hand-searched the reference list of the selected publications to retrieve additional relevant publications. The search string included Adaptive, OR Flexible, OR Tailored, AND Internet, AND Interventions, AND Mental Health (see Multimedia Appendix 1 for detailed search string). Each term included medical subject headings, and the search was done on full-text papers. The search was limited to all papers published in English from January 2000 to January 2020. The database searches and subsequent review were performed by the same two authors (SKM and JDW) independently in a double-blind process.

### Eligibility Criteria

We included studies in which the articles met the following inclusion criteria: (1) discussed an intervention delivered through the internet (web- or mobile-based), (2) attempted to provide adaptive (dynamic, tailored, flexible) interventions by using adaptive strategies, (3) targeted a mental health disorder defined by the Diagnostic and Statistical Manual of Mental Disorders, Fifth Edition (DSM-5) [38], and (4) was published between January 2000 and January 2020. No data restrictions were imposed. The following exclusion criteria were used: (1) not written in the English language, (2) not a full-text paper or published in the form of a short paper, extended abstract, abstract or poster, (3) designed as nonempirical findings such as opinion papers, reviews, editorials, letters, agendas, or study protocols, (4) dealt with adaptive technology in any domain other than mental health, or (5) was not about adaptive technology.

### Review Procedure

**Figure 1:**
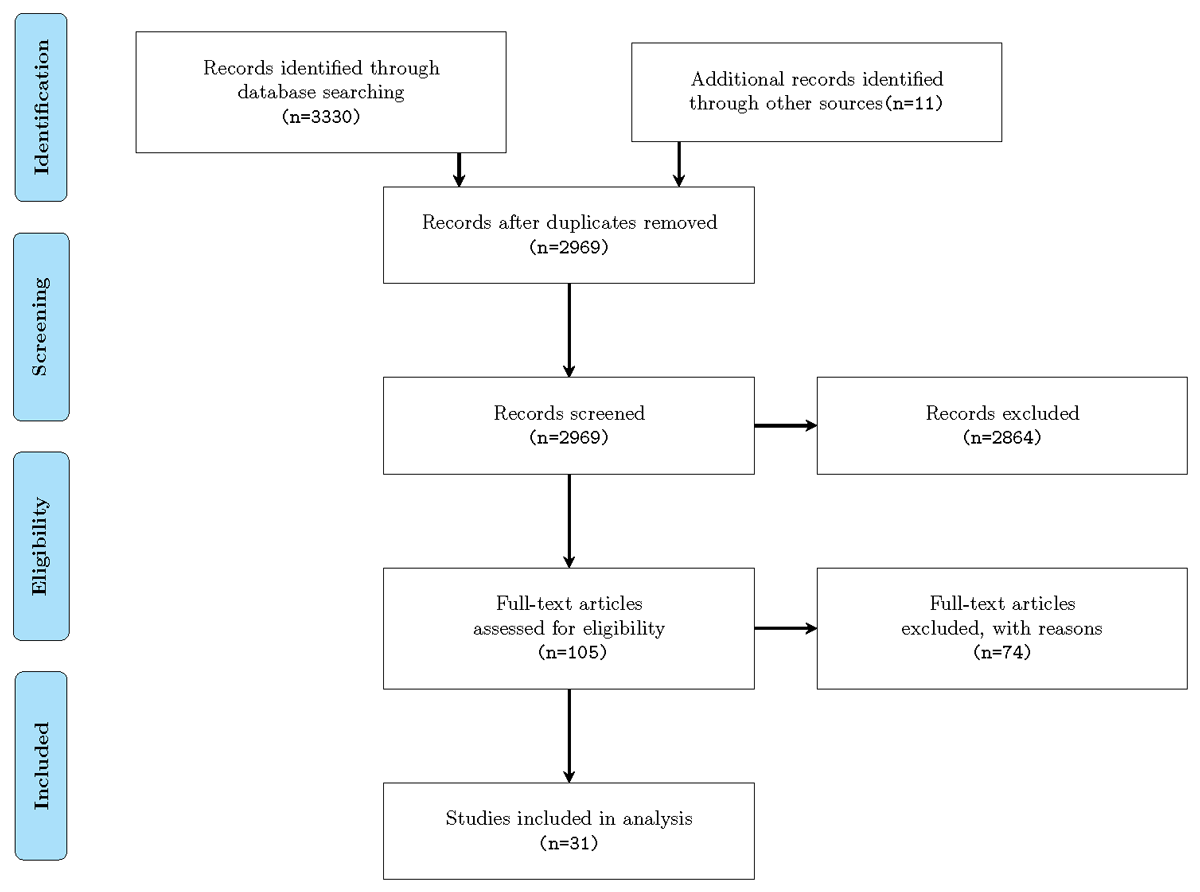
Preferred Reporting Items for Systematic Reviews and Meta-Analyses (PRISMA) flow diagram for this systematic review.

The selection of studies took place in three phases based on the review of the title, keywords, abstract, and full text. Title and abstract screening were carried out blinded for author, journal, and date of publication. Any doubtful papers were included in the next phase, and disagreement was resolved through discussion. After identifying 3341 relevant papers in the initial database search, 372 duplicate papers were removed, and 2969 unique papers remained. In the screening step, the resulting list of 2969 papers was reviewed independently by the same two authors according to inclusion and exclusion criteria. By reviewing the title, abstract, and keywords, 105 eligible papers were retrieved. Two main reasons for the substantial exclusions were (1) the search engine returned the results containing any of the search terms, although they were logically connected, and (2) most of the papers were related to mental health without any reference to IDPT. Full texts were evaluated to determine the eligibility of the remaining papers. The full texts of the 105 eligible papers were assessed independently by the same authors. Any discrepancies between the authors regarding the selection of the papers were resolved through discussion. In total, 74 papers were excluded in this round, and the selection process led to the inclusion of 31 papers, as illustrated in Figure 1. The most common reason for exclusion in this phase was that the publication did not discuss an intervention delivered via the internet. Other publications were excluded because they focused on other types of health care interventions without clear information about IA, user adherence, or adaptive strategies.

### Data Extraction and Synthesis

Data from the included studies were extracted, verified, and tabulated for review by the authors. From the selected studies, we chose to obtain the main adaptive elements, adaptive strategies used, adaptive dimension, and actor involved in adaptation. Multimedia Appendix 2 provides a detailed summary of the analysis of the 31 articles in the review. All of the articles in the analysis are listed in the references [11-14,24,32,39-63]. We evaluated all of the relevant studies based on the adaptive IDPT model previously described [28]. As mentioned in the study [28], we extracted the core components of the adaptive reference model, including adaptive elements, adaptive dimensions, IA, and adaptive strategies. The rest of our results are based on these core components.

### Data and Software Availability

For purposes of transparency and reproducibility of our study, we have published the resulting data, code, and procedures on GitHub [64]. The GitHub repository includes raw articles extracted from database searches, keyword formulation documents, preprocessed article lists, and a literate programming script used for data preprocessing, analysis, and visualization.

## Results

### Mental Health Illnesses Addressed

A significant number of the included studies addressed depression (n=11) and anxiety disorder (n=7), followed by general mental health issues (n=8), such as well-being, mindfulness, and goal achievement. Furthermore, some studies reported the use of adaptiveness in other areas such as insomnia (n=2), social psychology (n=1), attention deficit hyperactivity disorder (n=2), posttraumatic stress disorder (n=2), suicidality (n=2), and substance misuse (n=1). The full list of types of mental health problems addressed in the relevant studies is presented in Table 1.

**Table 1:** Types of mental illness for which an adaptive system was built.

Mental illnesses	Study references
Depression	Tsiakas et al, 2015 [40]
	Levin et al, 2018 [41]
	Burns et al, 2011 [50]
	Rebar et al, 2016 [51]
	Malins et al, 2020 [54]
	Van Gemert-Pijnen et al, 2014 [56]
	Lillevoll et al, 2014 [24]
	Achtyes et al, 2015 [57]
	Wallert et al, 2018 [58]
	Kop et al, 2014 [61]
	D’Alfonso et al, 2017 [63]
Anxiety disorder	Tsiakas et al, 2015 [40]
	Levin et al, 2018 [41]
	Walter et al, 2007 [45]
	Batterham et al, 2017 [48]
	Malins et al, 2020 [54]
	Achtyes et al, 2015 [57]
	Wallert et al, 2018 [58]
Insomnia	Forsell et al, 2019 [59]
	Erten-Uyumaz et al, 2019 [60]
Substance use	Batterham et al, 2017 [48]
Suicidality	Delgado-Gomez et al, 2016 [42]
	Batterham et al, 2017 [48]
Social psychology	Rachuri et al, 2010 [62]
Bipolar disorder	Dodd et al, 2017 [14]
Stress	Konrad et al, 2015 [47]
Posttraumatic stress disorder	Tielman et al, 2019 [43]
	Eisen et al, 2016 [46]
Smoking cessation	Lagoa et al, 2014 [44]
Attention deficit hyperactivity disorder	Nahum-Shani et al, 2012 [12]
General mental health	Iorfino et al, 2019 [39]
	Bannink et al, 2012 [11]
	Berrouiguet et al, 2018 [49]
	Ketelaar et al, 2014 [52]
	Coyle et al, 2010 [53]
	Kitagawa et al, 2020 [13]
	van Os et al, 2017 [55]
	van de Ven et al, 2017 [32]

### Intervention Platform

Based on our findings, the communication media used to administer internet-facilitated interventions to patients can be classified into three categories: web apps, mobile apps, and computer games. A significant number of the included studies were based on web apps [11,14,24,39,43,49,50,52,54,56,63], followed by mobile apps [13,32,41,49,50,55,60,61] and a game-based intervention [53]. There was only one paper that applied both web and mobile technologies for internet-based intervention. However, a lot of studies did not report the mode of delivery.

### IA

IA is concerned with the art and science of organizing and labelling components of web apps, intranets, software, and online communities to enhance their usability and accessibility. IA plays a vital role in web app development, and a good architecture can improve the ability of employees and customers to find information and decrease the app’s maintenance cost [28]. Finding the type of IA used in IDPT systems and their relevancy to treatment outcomes is one of the research questions of this study. However, a significant number of studies (20/31, 65%) did not report the type of IA they used in their IDPT system. Based on the IA of the intervention reported in the 31 reviewed articles, 4 (13%) studies reported the use of tunnel-based IA, and 3 (10%) studies reported the use of matrix IA and hierarchical IA. The full list of types of IA used in the relevant studies is presented in Table 2.

**Table 2:** Types of information architecture used in the reviewed studies.

Information architectures	Study references
Tunnel-based IA	Iorfino et al, 2019 [39]
	Konrad et al, 2015 [47]
	Batterham et al, 2017 [48]
	Kitagawa et al, 2020 [13]
Hybrid IA	D’Alfonso et al, 2017 [63]
Matrix IA	Levin et al, 2018 [41]
	Lagoa et al, 2014 [44]
	Van Gemert-Pijnen et al, 2014 [56]
Hierarchical IA	Tielman et al, 2019 [43]
	Bannink et al, 2012 [11]
	Berrouiguet et al, 2018 [49]
Not clear/not reported	Coyle et al, 2010 [53]
	Tsiakas et al, 2015 [40]
	Delgado-Gomez et al, 2016 [42]
	Walter et al, 2007 [45]
	Bannink et al, 2012 [11]
	Burns et al, 2011 [50]
	Rebar et al, 2016 [51]
	Ketelaar et al, 2014 [52]
	Malins et al, 2020 [54]
	Kitagawa et al, 2020 [13]
	van Os et al, 2017 [55]
	Lillevoll et al, 2014 [24]
	Dodd et al, 2017 [14]
	Achtyes et al, 2015 [57]
	Wallert et al, 2018 [58]
	Forsell et al, 2019 [59]
	Erten-Uyumaz et al, 2019 [60]
	Kop et al, 2014 [61]
	van de Ven et al, 2017 [32]
	Rachuri et al, 2010 [62]

The analysis of Table 2 answers our first research question. Most of these IAs fall into four categories: (1) tunnel-based design, (2) matrix design, (3) hierarchical design, and (4) hybrid design. A previous study [65] showed that 90% of the available IDPT systems used a tunnel-based design, where users navigate sequentially to search for information. A tunnel-based design is analogous to watching TV series, reading textbooks, attending academic classes, or attending multiple clinical sessions. An argument for tunnel-based design is that the experience is less likely to overwhelm users with information and options [66]. The tunnel-based design is probably also the default IA design alternative in many projects and is the easiest to implement.

### Adaptive Elements

Adaptive elements are the main components that are personalized for the user. As reported in a previous study [28], the main adaptive elements can be intervention content, design, assessment tests, IA, content presentation, content complexity, content recommendation, user interface (such as navigation system, search engines), feedback, notifications/reminders/alerts, behavioral activities, exercises, and reporting/dashboards. We report the full list of adaptive elements found in the relevant studies in Table 3.

Numerous studies (9/31, 29%) reported adapting the content of the intervention. However, most of these studies did not explicitly report the type of content, level of complexity, or modality (audio, video, presentation, pictures, assignments, activities, and assessments). Knowledge of the modalities of the content and their associated complexity provides insight into how interventions could be adapted and personalized for patients.

Another notable observation is that several studies (11/31, 35%) used feedbacks as adaptive elements. Numerous studies described the process of adaptive feedback in different forms, including sending personalized motivational messages [43], tailored messages by therapists [11,13,54], and providing general support [14]. In contrast, a few studies aimed to adapt reminders and alerts by sending an email or SMS text message or making a phone call [24,48,50]. Only 2 studies targeted the adaptation of exercises [41,47] and 1 study targeted the adaptation of behavioral activities [60]. We identified a total of 7 papers (7/31, 23%) that adapted assessment tests or psychometric assessment tests [39,42,45,46,55-57].

**Table 3:** Types of adaptive elements identified from the relevant studies.

Main adaptive elements	Study references
Intervention content	Iorfino et al, 2019 [39]
	Lagoa et al, 2014 [44]
	Batterham et al, 2017 [48]
	Rebar et al, 2016 [51]
	Coyle et al, 2010 [53]
	Nahum-Shani et al, 2012 [12]
	Van Gemert-Pijnen et al, 2014 [56]
	D’Alfonso et al, 2017 [63]
	Kop et al, 2014 [61]
Content presentation	Iorfino et al, 2019 [39]
Feedback message, support	Iorfino et al, 2019 [39]
	Tielman et al, 2019 [43]
	Bannink et al, 2012 [11]
	Batterham et al, 2017 [48]
	Burns et al, 2011 [50]
	Ketelaar et al, 2014 [52]
	Malins et al, 2020 [54]
	Kitagawa et al, 2019 [13]
	Van Gemert-Pijnen et al, 2014 [56]
	Dodd et al, 2017 [14]
	van de Ven et al, 2017 [32]
Assessment tests	Iorfino et al, 2019 [39]
	van Os et al, 2017 [55]
	Van Gemert-Pijnen et al, 2014 [56]
	Achtyes et al, 2015 [57]
	Delgado-Gomez et al, 2016 [42]
	Walter et al, 2007 [45]
	Eisen et al, 2016 [46]
Behavioral activities (sleep pattern)	Erten-Uyumaz et al, 2019 [60]
Reminder messages (SMS text messages, emails, phone calls)	Burns et al, 2011 [50]
	Lillevoll et al, 2014 [24]
	Batterham et al, 2017 [48]
Exercises	Levin et al, 2018 [41]
	Konrad et al, 2015 [47]
Reports	Iorfino et al, 2019 [39]
	Burns et al, 2011 [50]
Not clear	Tsiakas et al, 2015 [40]

Table 3 presents the list of the primary adaptive elements in the adaptive IDPT systems. The list includes (1) intervention content, (2) content presentation, (3) feedback messages, (4) assessment tests, (5) behavioral activities (sleep pattern), (6) reminder messages (email, SMS text messages, phone calls), (7) exercises, and (8) reporting (dashboard for the patients and the therapists). The central concept of adaptiveness is to create different levels of these adaptive elements and provide these elements based on a personalized profile. For example, if a person watches videos more than they listen to audio, read text, or view slides, then based on the principle of adaptiveness, it makes sense to present upcoming interventions in a video format.

### Dimensions of Adaptation

The way an adaptive system changes its behaviors depends on a multitude of factors: (1) users’ data and preferences, (2) goals of the intervention, (3) measures, (4) adaptation actors, and (5) adaptation strategies. We refer to these aspects as the dimensions of the adaptive IDPT system [28]. Table 4 presents a list of adaptive dimensions extracted from the included studies.

**Table 4:** Dimensions considered for adaptation in the relevant studies.

Adaptation dimensions		Study references
User data and preferences (user context, needs, and location)		Iorfino et al, 2019 [39]
		Delgado-Gomez et al, 2016 [42]
		Tielman et al, 2019 [43]
		Lagoa et al, 2014 [44]
		Walter et al, 2007 [45]
		Eisen et al, 2016 [46]
		Van Gemert-Pijnen et al, 2014 [56]
		Dodd et al, 2017 [14]
		Forsell et al, 2019 [59]
		Erten-Uyumaz et al, 2019 [60]
		Kop et al, 2014 [61]
		van de Ven et al, 2017 [32]
		Rachuri et al, 2010 [62]
		D’Alfonso et al, 2017 [63]
**Measures**		
	Psychometric tests/screening	Tsiakas et al, 2015 [40]
		Levin et al, 2018 [41]
		Bannink et al, 2012 [11]
		Batterham et al, 2017 [48]
		Berrouiguet et al, 2018 [49]
		Burns et al, 2011 [50]
		Rebar et al, 2016 [51]
		Ketelaar et al, 2014 [52]
		Coyle et al, 2010 [53]
		Malins et al, 2020 [54]
		Nahum-Shani et al, 2012 [12]
		Kitagawa et al, 2019 [13]
		Van Gemert-Pijnen et al, 2014 [56]
		Achtyes et al, 2015 [57]
		Wallert et al, 2018 [58]
	User behavior analysis based on interaction data	Berrouiguet et al, 2018 [49]
		Burns et al, 2011 [50]
Goals of intervention		Konrad et al, 2015 [47]
Not clear		van Os et al, 2017 [55]
		Lillevoll et al, 2014 [24]

The relevant studies were mainly grouped into two clusters based on the choice of adaptive dimensions: user preferences (13/31, 42%) or outcome measures (14/31, 45%). Only 1 study used a goal-based adaptive dimension. Among the studies using user preferences, some studies [32,42-46,62] used user context, while some studies used user location [32,62] to adapt interventions. The studies based on outcome measures used either psychometric tests or user behavior analysis based on interaction data to measure the performance outcome.

### Adaptive Strategies

The adaptive strategy indicates the techniques used to tailor the intervention. In a recent study [28], four significant clusters of adaptive approaches were identified, namely rule-based adaptation, predictive algorithm-based (such as machine learning) adaptation, goal-driven adaptation, and adaptation through a feedback loop. Similar to the study [28], we identified the following adaptive strategies, presented in Table 5, in the reviewed studies.

**Table 5:** Types of adaptive strategies found in the relevant studies.

Types of adaptive strategies	Study references
Rule-based strategies	Iorfino et al, 2019 [39]
	Tsiakas et al, 2015 [40]
	Levin et al, 2018 [41]
	Delgado-Gomez et al, 2016 [42]
	Tielman et al, 2019 [43]
	Walter et al, 2007 [45]
	Eisen et al, 2016 [46]
	Bannink et al, 2012 [11]
	Konrad et al, 2015 [47]
	Batterham et al, 2017 [48]
	Rebar et al, 2016 [51]
	Ketelaar et al, 2014 [52]
	Coyle et al, 2010 [53]
	Malins et al, 2020 [54]
	Nahum-Shani et al, 2012 [12]
	Kitagawa et al, 2019 [13]
	van Os et al, 2017 [55]
	Van Gemert-Pijnen et al, 2014 [56]
	Lillevoll et al, 2014 [24]
	van de Ven et al, 2017 [32]
Predictive algorithm- or machine learning–based strategies	Tsiakas et al, 2015 [40]
	Lagoa et al, 2014 [44]
	Berrouiguet et al, 2018 [49]
	Burns et al, 2011 [50]
	Nahum-Shani et al, 2012 [12]
	Wallert et al, 2018 [58]
	Rachuri et al, 2010 [62]
	Erten-Uyumaz et al, 2019 [60]
	Kop et al, 2014 [61]
Recommendation-based strategies	D’Alfonso et al, 2017 [63]
General or unclear strategy	Dodd et al, 2017 [14]
	Achtyes et al, 2015 [57]

The list of strategies includes rule-based strategies, predictive algorithm-based strategies, and recommendation-based strategies. As expected, a significant number of studies (20/31, 65%) used some form of rule-based adaptation mechanism. For example, some studies [42,45,46] used item-response theory [8] to tailor psychometric tests. The primary motivation toward adapting psychometric tests is to extract essential information from patients without asking them too many questions. The study by Van Gemert-Pijnen et al [56] applied user behavior analysis by inspecting web-log data and combined it with a rule-based engine to adapt the intervention. The same study concluded that pattern recognition can be a useful tool to tailor interventions based on usage patterns from earlier lessons [56]. With the hype of data science, several studies [12,40,44,49,50,58,60-62] attempted to use some form of predictive algorithm to adapt the intervention. While some studies did not report the outcome of the overall study [40,49], most of them reported that the use of predictive algorithms had a positive effect on the adaptation of interventions [12,44,50,58,60-62]. However, these studies concluded that further research is required to study the effectiveness of performance outcomes.

### Efficacy of Treatment Outcomes

In general, this systematic review shows that tailoring interventions according to patients’ needs and preferences has a positive effect on user adherence and hence treatment outcomes. Several studies reported that the personalization of interventions [13,32,39,41,43,47,50-52,54,56,59] and assessments [42,45,46] increased user adherence. Similarly, a study by Coyle et al [53] reported that a personalized system provided a higher degree of user satisfaction. However, some studies reported a lack of noticeable improvement in treatment outcomes. For instance, a study by Batterham et al [48] reported that there was no significant difference between tailored and static versions in the effectiveness of treatment or adherence. However, the same study reported that participants in the tailored conditions were more satisfied than those in the control conditions [48]. Lillevoll et al [24] made a similar conclusion, reporting that tailoring of feedback and dispatching weekly email reminders did not improve the intervention outcomes or user adherence. Given the scenario with two different clusters of results, further research and clinical trials are required to comprehend how user adherence and personalization of interventions are correlated.

## Discussion

### Key Findings

A total of 3341 studies were initially identified based on the inclusion criteria. Following a review of the title, abstract, and full text, 31 studies that fulfilled the inclusion criteria remained, most of which attempted to tailor interventions for mental illnesses. Approximately 68% (21/31) of the studies had a first author with a health care background. The most common adaptive elements were feedback messages to patients from therapists (11/31, 35%) and intervention content (9/31, 29%). However, how these elements contribute to the efficacy of IDPT in mental illness was not reported. The most common IA used was tunnel-based IA (4/31, 13%), while many studies (20/31, 65%) did not report the IA used. The rule-based technique was the most common adaptive strategy used in these studies (20/31, 65%). All the studies were broadly grouped into two adaptive dimensions based on user preferences or using performance measures such as psychometric tests.

### Intervention Platform

**Figure 2:**
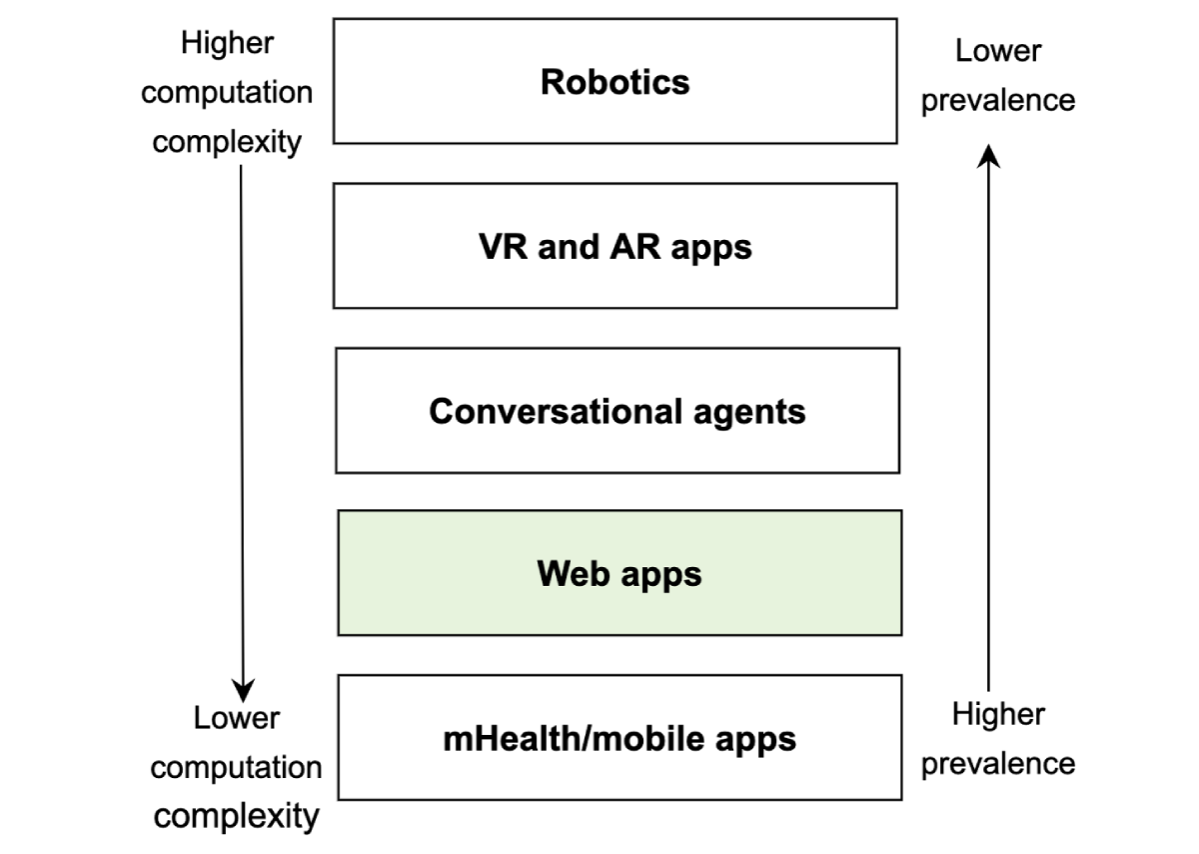
Mode of delivery for internet-delivered psychological treatments (IDPTs). AR: augmented reality; mHealth: mobile health; VR: virtual reality.

Our findings show that web apps, mobile apps, and computer games are the primary platforms used to facilitate interventions. Apart from these, other communication media include robotics, virtual reality (VR) [67], augmented reality (AR), conversational agents, or chatbots [68]. A key finding in the literature study was that most of the IDPTs were made available on mobile apps [69] and web-based apps. This is as expected, as these are the most prevalent platforms used for personal computing. Smartphones contain a plethora of sensors and other data sources that inform aspects of users’ well-being, context, activities, behaviors, and intentions. However, only a few attempts have been made to provide IDPT using conversational agents [68], or VR or AR apps. As depicted in Figure 2, higher placement in the chain indicates higher computational complexity but lower prevalence. Conversely, lower placement in the chain indicates lower computational complexity but higher prevalence. The most obvious explanation for the selection of web and mobile apps for intervention platforms is their prevalence. It makes the most sense to develop for platforms that are being used the most.

### IA

Although a significant number of the studies failed to report which IA was used in their IDPT system, IA is still present in all software systems. Understanding the IA of a system helps a user to store, find, and interpret information readily, as IA is the design principle that is applied to making information discoverable and understandable. Finding the underlying IA of the IDPT system can help in making systems accessible and discoverable for end users, and knowledge about information design, structure, organization, and labelling can facilitate the development and evaluation phase. As explained in a previous study [70], IA consists of three major components: user, content, and context. Hence, IA helps to discover content for users based on their context. Assisting users with the correct piece of information they are looking for can increase user adherence by reducing bounce rate [71] and hence improve treatment outcomes. While our findings show most of the current adaptive IDPT systems are tunnel-based and don’t promote personalization, we promote the use of hierarchical IA or hybrid IA in order to present useful information according to patients’ needs and argue that the latter designs offer richer opportunities for adaptive IDPT systems.

### Adaptive Elements

The primary context for building adaptive IDPT systems is to assist patients suffering from mental health disorders to learn about and recover from their illnesses. An IDPT system provides this information by using different media and elements such as text, video, audio, pictures, presentations, feedback, reminders, reports, and others. These elements have their format, structure, metadata, volume, and dynamism (such as frequency of updates). It is essential to understand these elements (contents described by Pakkala et al [71]) and the associated attributes in order to deliver the correct content to the right user in their current context. For example, understanding the complexity of the text describing sleep disorders can help to personalize the content based on the educational background of the patients. A general assumption is that an educated person can comprehend more complex language and medical terms than a nonspecialist. For a nonspecialist, it is more valuable to present the same content in terms of animated videos or pictures.

### Adaptive Dimensions

The adaptive dimension provides the context for an adaptive IDPT system to tailor its behavior. A common way to tailor the behavior of the IDPT system is based on input regarding user preferences, measures (psychometric tests, user behavior analysis, and others), and goals, as shown in Table 4. In the context of user preferences, a study by Yardley et al [72] outlined that users can feel overwhelmed by the quantity and complexity of information presented to them. Hence, it is advisable to meet the needs of different users based on their needs and preferences. Personalization is critical mainly because users have different cognitive skills, educational backgrounds, content format preferences, comprehension capabilities, and other qualities. A similar conclusion was made by Sundar and Marathe [73]. Their study identified two types of users: those influenced more by the affordance of agency (power users) and those influenced by the relevancy of the resulting content. The results of the study revealed that nonpower users preferred personalized content, whereas power users rated content quality higher when the website had a customizable feature. Similar to user preferences, other dimensions of adaption are performance measures such as the use of psychometric tests (eg, Patient Health Questionnaire-9 [74] for depression), user behavior analysis, and others.

### Adaptive Strategies

Adaptive strategies provide a mechanism to present the right content to the right people based on their needs and preferences. While our review findings reveal that the rule-based adaptive strategy is the most widely adopted practice, other strategies, especially machine learning, are becoming highly prevalent. Given the premise that we can capture every digital footprint of a user, resulting in a complex and comprehensive data set, there is a possibility of using sophisticated machine learning or deep learning algorithms on the one hand, but it also raises an essential question about privacy on the other. In general, to build an adaptive IDPT system, it is crucial to understand which adaptive strategies can be used. Based on the selected strategies, one needs to collect and store the data. No matter which adaptive strategy is chosen, the adaption in an IDPT system is an iterative cycle where data is collected and preprocessed; preprocessed data are then analyzed and, based on the results of the analysis, an action is taken to tailor the intervention. However, how the data are analyzed and the result is extracted affect the way an IDPT system is developed, the choice of IA, the method of data storage, and other parameters.

### Challenges in Contemporary IDPT Systems

Although the integration of health care systems has emerged as a policy for several health care agencies, there is a large gap between current policy, program implementation efforts, and evidence for health care integration. The results of our review led us to list the following challenges in current IDPT practice.

#### Lack of Standard Taxonomy in IDPT

There is a lack of a standardized definition of the health care system and proper taxonomy to allow the grouping of similar interventions. As mentioned in the introduction, the use of nonstandard terms to refer to the same system causes inconsistencies and makes it hard to draw conclusions. Based on this challenge, several researchers [75-77] have made an effort to formalize the health care system to support interoperability [76], such as the Fast Healthcare Interoperability Resources (FHIR) created by the Health Level Seven International (HL7) health care standards organization [78] and others. If there is a standard followed by researchers for building adaptive IDPT systems, it will become easier to learn from their findings and extend the current understanding to improve treatment outcomes. 

#### Scientific Foundation

The outcome of trials of IDPT systems has demonstrated comparable results as face-to-face therapies. However, despite considerable attention to IDPTs, user adherence is low, and there is remarkably less literature on the underlying science of the field of IDPT system design and development [79]. Many pieces of literature claim IDPT to be based on psychoeducation that helps in the modification of behavior change and symptom improvement. While this assumption may be correct, the underlying science behind how psychoeducation about particular symptoms enhances behavior modifications and symptom improvement is less evident in the literature. Lack of a scientific foundation behind IDPT systems may be the reason behind users’ lack of trust toward interventions [21-23] and hence lack of adherence. In the same way, an IDPT system is an application software that follows an IA (see Table 2) and design patterns [80]. The application software such as IDPT are well formalized and studied in the research community. Lack of such reporting makes it challenging to conclude how adaptive elements or the IA influence the outcome of an intervention.

#### Ethical and Safety Issues Associated with Predictive Adaptive Strategies

Technology has matured to the point where several researchers envision the creation of automated, adaptive IDPT systems that work without much human involvement. However, there are controversies between what is possible and what is acceptable in adaptive systems. Hence, it requires careful consideration of both ethical and legal issues; focusing solely on technological and operational perspectives can lead to low value or utility for patients. As a result, both information and communication technology (ICT) researchers and medical practitioners must consider the capabilities, limitations, and needs of patients when designing adaptive systems. The primary objective of the adaptive IDPT system is to tailor the intervention based on user needs or any other adaptive dimensions. The adaptive IDPT system can understand the user’s needs by creating detailed user profiling. User profiling includes storage of the patient’s previous diagnosis, sensitive personal information, as well as the current status. Moreover, to maximize the benefits of data-driven adaptiveness, the adaptive IDPT system needs to store interaction data, including the time of login, the frequency of login, and the interaction with the system at the granular level (clicks, keystrokes). For example, the study by Van Gemert-Pijnen [56] analyzed the log data in order to understand the use of the content. Storing such user interaction data requires proper user consent on the one hand and directly deals with the privacy of the patient on the other. Hence, it is one of the critical challenges in the development of an adaptive IDPT system. It requires further research into the problem of storing user interaction data securely. For example, many psychological interventions aim to characterize patients’ symptoms based on their mobile phone usage. This type of study is possible because mobile phones come with built-in sensors and standard application programming interfaces to measure and collect patients’ data, including mobility patterns, physical activities, crowd density, time spent indoors versus outdoors, and locations. Although these capabilities are possible with the advances in ubiquitous computing, they deal directly with privacy and ethical issues.

### Implications and Future Directions

It is not easy to predict how technologies will develop over time and whether these technologies will continue adapting to clinical use. However, based on the results of this systematic review, we outline some implications and future directions in the field of IDPT system development and innovation.

#### Implications for ICT Researchers

With an increasing trend in user adherence toward internet-delivered treatments on the one hand and the prevalence of the internet of things (IoT), with growth in ambient intelligence technology, on the other, there is an expectation that the IDPT system will flourish over time. A plethora of health care interventions delivered via the internet have a similar format, as most of them are based on psychoeducation. All such interventions attempt to create adaptive elements (see Table 3) and attempt to tailor these elements based on adaptive dimensions (see Table 4) using adaptive strategies (see Table 5). Hence, it makes sense to create a conceptual framework that can be utilized in several health care domains. Moreover, the creation of domain-specific languages for incorporating adaptive health care interventions is also required. We also need better dashboard tools that help therapists and other medical practitioners to comprehend patients’ status better and adapt their interventions based on their engagement with the interventions.

We analyzed the state-of-the-art studies concerned with adapting psychological interventions. The analysis yielded the answers to the most critical questions, including (1) what are the essential elements that therapists wish to tailor? (2) what are the main dimensions in which these elements can be tailored to meet patients’ needs? and (3) what are the primary adaptive strategies used to trigger adaptation in those dimensions? Findings from the analysis helped to identify the essential variables that are associated with an adaptive system. As McGaghie et al [81] outlined, once ICT researchers and developers know the essential variables, they can utilize these findings to create a conceptual framework that sets the stage for the presentation of a particular problem, which in this case is the creation of an adaptive IDPT system. Further, ICT researchers and developers can validate the conceptual framework by building domain-specific language.

#### Implication for Health Care Researchers

While the current research evidence is fragmented about the benefit of an adaptive IDPT system on treatment outcomes, this review suggests that adaptive IDPT systems can benefit people with mental health issues in providing personalized psychoeducation. Such an education will help mental health patients to manage their illness. In addition, a high number of health care researchers have published about adaptive interventions, as shown in Figure 2. This indicates that both health care researchers and computer researchers believe that adaptive interventions are an essential phenomenon to accelerate user adherence. However, tailoring the feedbacks given by therapists or providing reminders are simple forms of adaptation (see Table 3). The intervention can be adapted for several dimensions, including user preferences, outcome measures, and different adaptive strategies [28], with the amalgamation of ambient technology. Hence, cooperation between ICT and health care researchers is essential to develop an adaptive IDPT system. While more randomized controlled trials are required to validate the effectiveness of adaptive treatments, the results of this review show sufficient evidence to suggest that the adaptation of mental health interventions can enhance user adherence and treatment outcomes.

#### Implication for Computer Science Research

The development of an adaptive IDPT system that increases user adherence and treatment outcomes requires more extensive research to establish clinical appropriateness. Given the potential benefit of the IDPT system for cost-effective delivery to the far-reaching population, further research should be conducted on how to personalize adaptive strategies. Furthermore, reporting back to the research community is the part of any discipline of transparency that keeps studies honest and accountable. In addition, it fits into the broader responsibilities of scientists to communicate their work and foster public understanding. Such understanding can be used by other researchers to gain insight into new research directions.

### Future Work

An immediate future task involves the creation of a conceptual framework for adaptive IDPT systems. In addition to this, we envision the development of domain-specific language that can model such an adaptive IDPT system. Furthermore, it is imperative from the review that there is a need for a comprehensive visual dashboard for therapists and patients where they can receive the intervention, monitor their symptoms, and manage their illness.

### Limitations

Given that the health ICT literature is quite diverse and extensive, the current study focused exclusively on internet-delivered interventions for mental health morbidities. Notwithstanding this limitation, this paper highlights the significance of the continued study of this intervention method. Another limitation is that our literature exploration only encompassed articles in the English language; therefore, it is plausible that some research conducted in other parts of the world and published in other languages were missed. A third limitation pertains to IDPT apps developed by industry that were not accessible for review. Hence, we have less knowledge about the adaptive elements involved in their architecture.

### Conclusions

Adaptive psychological interventions tailor the type of content or tasks to individuals based on their needs and preferences in order to improve saliency and intervention efficacy. This systematic review describes the investigation and analysis of existing studies about adaptive psychological intervention delivered through the internet. The study outlines the main elements used in the process of adaptation, the IA used in the adaptive systems, the main dimensions of adaptation, and the main adaptive strategies. Based on these findings, we envision the development of a conceptual framework that researchers and clinicians can utilize to build adaptive models of several health care interventions.

The findings of our review indicate the use of web-based and mobile apps to deliver mental health interventions, such as for depression (most studied), anxiety, and others. However, a number of these studies did not report the IA used in their system, and those that did report mostly used tunnel-based systems. Similarly, several studies used rule-based adaptive strategies to adapt intervention based on performance measures such as psychometric tests. Feedback messages, reminders, and support were the most used adaptive element. Further study is required to explore the role of IA, adaptive elements, adaptive dimensions, and adaptive strategies in building a successful IDPT system. Knowledge about these core elements of the adaptive IDPT system can serve to create a conceptual framework that can be used for several health care interventions.

## Appendix

Multimedia Appendix 1 Search terms that were used to execute the search in different databases.

Multimedia Appendix 2 The main table that helped to extract the adaptive elements, adaptive strategies, information architecture, and other information about the internet-delivered psychological treatment system.

## Abbreviations

AR: augmented reality
DSM-5: Diagnostic and Statistical Manual of Mental Disorders, Fifth Edition
FHIR: Fast Healthcare Interoperability Resources
HL7: Health Level Seven International
IA: information architecture
ICT: information communication technology
IDPT: internet-delivered psychological treatment
PRISMA: Preferred Reporting Items for Systematic Reviews and Meta-Analyses
VR: virtual reality
