# Supplementary material for: Adaptive Elements in Internet-Delivered Psychological Treatment Systems: Systematic Review
Source: J Med Internet Res. 2020 Nov 27;22(11):e21066. doi: 10.2196/21066 (PMC7732710; doi:10.2196/21066)
Supplement: Multimedia Appendix 1 [file jmir_v22i11e21066_app1.pdf]

Multimedia Appendix 1: Adaptive Element in Architecture of Internet-Delivered Treatments systems to improve user adherence: Systematic Review

Suresh Kumar Mukhiya (skmu@hvl.no) et al.

Search String

```
 [(adaptive OR tailored OR flexible OR personalized OR customized)
AND (Internet OR computer OR online OR mobile OR smartphone OR digital
OR electronic OR ICBT) AND (intervention OR treatment OR assessment)
AND (mental health OR mental illness OR psychiatric illness OR psychiatric
disorder OR psychological disorder)
```

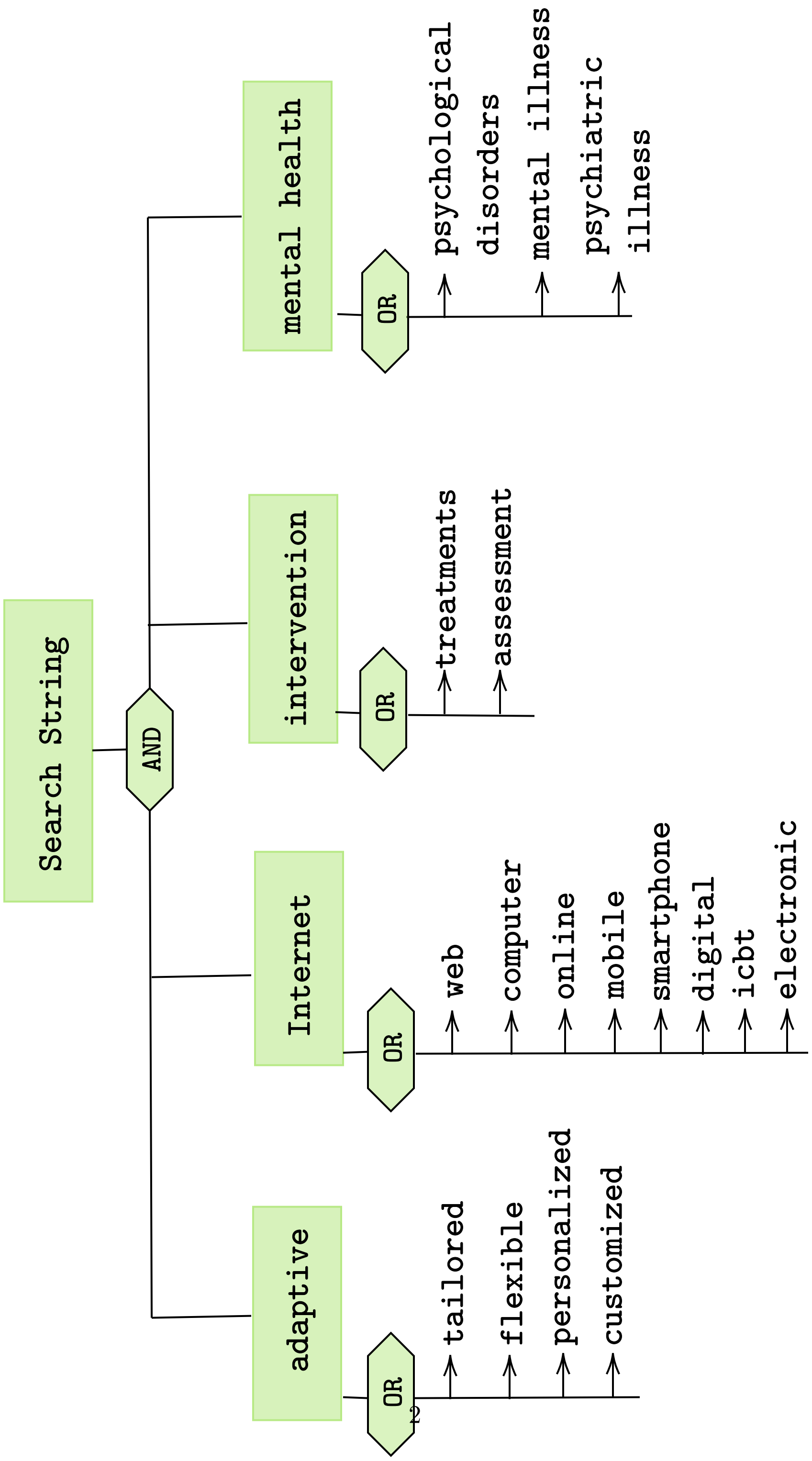

Figure 1: Search String Keywords Tree
